# Brain Oscillatory Correlates of Visual Short-Term Memory Errors

**DOI:** 10.3389/fnhum.2019.00033

**Published:** 2019-02-13

**Authors:** Igor Mapelli, Tolga Esat Özkurt

**Affiliations:** Neurosignal Laboratory, Graduate School of Informatics, Middle East Technical University, Ankara, Turkey

**Keywords:** working memory, false memory, memory errors, neural oscillations, visual, EEG

## Abstract

Brain dynamics of memory formation were explored during encoding and retention intervals of a visual working memory task. EEG data were acquired while subjects were exposed to grayscale images of widely known object categories (e.g., “luggage,” “chair,” and “car”). Following a short delay, two probes were shown to test memory accuracy. Oscillatory portraits of successful and erroneous memories were contrasted. Where significant differences were identified, oscillatory traits of false memories (i.e., when a novel probe item of the same category is recognized as familiar) were compared with those of successful and erroneous memories. Spectral analysis revealed theta (6–8 Hz) power over occipital channels for encoding of successful and false memories that was smaller when compared to other types of memory errors. The reduced theta power indicates successful encoding and reflects the efficient activation of the underlying neural assemblies. Prominent alpha-beta (10–26 Hz) activity belonging to the right parieto-occipital channels was identified during the retention interval. It was found to be larger for false memories and errors than that of correctly answered trials. High levels of alpha-beta oscillatory activity for errors correspond to poor maintenance leading to inefficient allocation of WM resources. In case of false memories, this would imply necessary cognitive effort to manage the extra semantic and perceptual load induced by the encoded stimuli.

## Introduction

Working memory (WM) is a fundamental constituent of the cognitive system. It encompasses short-term memory (STM), i.e., the temporary storage unit and all the processes responsible for monitoring, maintaining, and manipulating task-relevant information over a brief period of time (Baddeley, 2003). Visual WM (VWM) refers to visual sensory information that is encoded into internal neural representations and subsequently maintained by WM processes.

Visual working memory, like other critical memory components, is susceptible to distortions. Investigating memory failures with respect to successful performances may help to understand the underlying neural mechanisms in memory formation. False memories, unlike common errors, may arise due to pre-existing semantic associations (Koutstaal et al., 2003) and/or prototypical perceptual features (Gutchess and Schacter, 2012) additionally elicited by the encoded sequence. Paradigms were devised to study false memories by introducing novel items similar to the encoded items (i.e., lures) sharing perceptual or semantic properties and trapping subjects into erroneous responses (DePrince et al., 2004; Brainerd and Reyna, 2005). Most of the studies on visual false memories were conducted in the domain of long-term memory (LTM) (Israel and Schacter, 1997; Koutstaal and Schacter, 1997; Seamon et al., 2000; Koutstaal et al., 2001; Jones et al., 2006; Baioui et al., 2012).

While the vast majority of research investigated false memories in LTM, some recent studies have explored them over short-term periods. As suggested by the aforementioned theoretical accounts based on LTM studies, additional LTM activations induced by the encoded set contribute to the occurrences of false memories. This assumption remains valid in the STM context where LTM activations may interfere with WM content. Typically, LTM investigations use longer lists of stimuli to be studied and longer delays between encoding and recognition sessions. An advantage of having trials that last only a few seconds is that participants can be continuously monitored throughout all three main intervals, i.e., encoding, retention, and recognition. Short-term false memory studies mostly investigated behavioral measurements (Coane et al., 2007; Atkins and Reuter-Lorenz, 2008; Flegal et al., 2010; Flegal and Reuter-Lorenz, 2014; Olszewska et al., 2015). There have been just a few studies examining BOLD responses (Atkins and Reuter-Lorenz, 2011; Iidaka et al., 2014) and electrophysiological characteristics (Chen et al., 2012; Melnik et al., 2017) in order to determine neural markers of these events. Specifically, in a STM Deese-Roediger-McDermott study using Chinese words to be encoded (Chen et al., 2012), the authors reported a prominent ERP N400 effect over frontal, central and parietal midline electrodes for correctly recognized probes when compared to that of false memories. In a modified Sternberg paradigm with short lists of words presented auditorily, Melnik et al. (2017) identified prominent alpha band activity in posterior regions corresponding to false memories induced by semantic interference. Furthermore, one fMRI study searched for neural correlates of false memory phenomenon in VWM, while subjects performed a modified delayed match-to-sample test with human faces as stimuli (Iidaka et al., 2014). The authors reported an active role played by the amygdala amid short-term false memory events.

Encoding and retention are two critical VWM phases, where improper neural activations may lead to memory failures. The initial factor influencing memory performance is the translation of the sensory input into VWM representations (Cohen et al., 2012; Killebrew et al., 2018). Though, successful performances do not solely depend on optimal encoding of the to-be-remembered information but also on the maintenance of it. The efficiency of memory processes relies on a tight synchronization of neural oscillations with a precision in the millisecond range (Lisman and Idiart, 1995; Klimesch, 1996; Buzsáki and Draguhn, 2004; Singer, 2009; Palva et al., 2010; Eriksson et al., 2015). Both electroencephalography (EEG) and magnetoencephalography (MEG), due to their higher temporal resolution, have been important tools for the investigation of oscillatory dynamics related to the encoding and retention of VWM.

A wide range of studies reported amplitude modulation of theta (for a review see Sauseng et al., 2010) and alpha (for a review see Jensen and Mazaheri, 2010 and Klimesch et al., 2011) band activities during VWM tasks. Intracranial EEG studies demonstrated the occurrence of theta oscillations in the human cortex during the encoding interval of a Sternberg task (Howard et al., 2003; Rizzuto et al., 2003). Raghavachari et al. (2001) reported event-related theta band activity gated at many sites widely dispersed over the cortex. The amplitude of theta oscillations increased sharply at the beginning of each trial of the Sternberg task and returned to baseline level only after the subject’s response. In a subsequent investigation conducted by the same group, the theta power increase was found to be mostly situated in the parieto-occipital and temporal cortical regions (Raghavachari et al., 2006). In another study by Sederberg et al. (2003), successful memory encoding of words was associated with a significant theta power increase predominantly located in the right temporal and frontal sites. It has been suggested that cortico-hippocampal feedback loops may drive theta activity into cortical regions (Buzsáki, 1996). This reflects the novel encoded information while maintaining cortical areas of interest into a state of resonance (Miller, 1991; Klimesch, 1996, 2000; Klimesch et al., 1997a; Mölle et al., 2002). Beyond hippocampal functions, frontal midline theta band activity that reaches its maximum power around the Fz electrode site, has been linked to sustained attention (Sauseng et al., 2007) and was found to be positively correlated with both WM load and cognitive effort (Gevins et al., 1997; Jensen and Tesche, 2002; Onton et al., 2005). For an in-depth review on human and animal studies pertaining to WM and the frontal midline theta activity, we refer the reader to Hsieh and Ranganath (2014).

Complementary to EEG theta power increase observed during encoding, studies also reported alpha power decrease (Klimesch, 1996, 1999; Mölle et al., 2002), which presumably reflects increased excitability of the involved cortical areas (Klimesch et al., 1997a; Stipacek et al., 2003; Lange et al., 2013). Conversely, high levels of alpha activity are associated with low neuronal excitability. For instance, when attention shifts toward external visual information, alpha band activity in occipital areas was shown to decrease (Worden et al., 2000; Sauseng et al., 2005) enhancing perceptual performance (Thut et al., 2006; Hanslmayr et al., 2007; Van Dijk et al., 2008). On the other hand, when attention is directed inward for maintenance of VWM internal representations, alpha power increases (Jensen et al., 2002; Tuladhar et al., 2007) preventing external interferences (Rihs et al., 2007; Foxe and Snyder, 2011). Studies using EEG/MEG source modeling provided further evidence in support of the inhibition-timing hypothesis by observation of alpha power increase over task-irrelevant regions during WM tasks (Haegens et al., 2010; Roux et al., 2012). Importantly, alpha frequency was shown to vary across individuals (Klimesch, 1999) and the peak frequency in occipital areas was reported to increase along with the cognitive load leaking in some cases into the beta band (Haegens et al., 2014). Inhibitory alpha power levels during WM maintenance were also reported to positively correlate with memory load (Jensen et al., 2002; Tuladhar et al., 2007). Less explored, cortical beta oscillations observed for visual tasks were suggested to reflect visual attention (Wróbel, 2000) and were associated with STM processes (Tallon-Baudry et al., 1999; Medendorp et al., 2007; Palva et al., 2011) hypothesized to support the endogenous reactivation of WM content (Spitzer and Haegens, 2017).

Functional magnetic resonance imaging (fMRI) studies have also contributed to the mapping of cortical regions associated with VWM. Reportedly, frontal and parietal BOLD activity reflected executive functions (Carpenter et al., 2000; Linden et al., 2003; Osaka et al., 2004; Brass et al., 2005; Yuan and Raz, 2014; Bettcher et al., 2016) and selective attention (Kastner and Ungerleider, 2000; Mayer et al., 2007; Gazzaley and Nobre, 2012). Among the different visual-related areas, sustained activity in temporal, and occipital regions reflected the maintenance of object representations (Grill-Spector et al., 2001; Kourtzi and Kanwisher, 2001; Bell et al., 2009). Moreover, two studies applied pattern classification techniques to obtain the BOLD activity from the visual cortex during the delay period of delayed discrimination tasks. They were able to predict, on a trial basis, which type of orientation (Harrison and Tong, 2009) and color (Serences et al., 2009) were held in VWM. These results supported the view that sensory cortical areas contribute to VWM retention of fine-tuned feature information (Pasternak and Greenlee, 2005).

To the best of our knowledge, oscillatory correlates associated with visual memory errors, including false memories specifically over short-term periods, have not been investigated, yet. The current study was conducted to explore the temporal dynamics of EEG oscillatory activity reflecting VWM performance. We aimed at identifying time-frequency windows and locations distinguishing successful and erroneous short-term memories of grayscale photos of commonly seen object categories (e.g., “luggage,” “chair,” and “car”). Each category was defined by a set of images sharing the general thematic information (*gist*) while differing in the details characterizing the individual items (*verbatim*) (Koutstaal and Schacter, 1997; Brainerd and Reyna, 2002). The analysis concentrated on low frequencies (4–32 Hz), particularly theta and alpha bands, as the aforementioned studies suggested that they played prominent roles in the encoding and maintenance intervals of VWM. Furthermore, we attempted to induce short-term false memories and looked for potential oscillatory markers differentiating them from other types of errors. To this end, we devised a challenging VWM task with the intent to maximize the rate of erroneous memory responses by the encoding of visual stimuli presented sequentially at a fast-pace.

## Materials and Methods

### Participants

A total of 40 volunteers partook in this study. Six participants were excluded: one due to technical problems, one reported to have given random answers due to drowsiness and four provided a selection between two alternatives whenever faced with the paired probes, not following the given instructions (see the experimental design provided in “Paradigm”). Hence, there remained 34 participants (mean age *M* = 24.88, *SD* = 4.77, 16 females) for the analysis.

Six subjects contributed only with behavioral data. For the remaining 28 subjects (mean age *M* = 23.54, *SD* = 3.77, 12 females), EEG data were also recorded. Eligibility criteria included right-handedness and no use of medications that may affect the central nervous system. All subjects reported normal or corrected-to-normal visual acuity. They were informed about the experimental procedure and provided written informed consent prior to data collection in accordance with the Declaration of Helsinki. The METU ethics committee approved all experimental procedures.

### Stimuli

**Figure 1:**
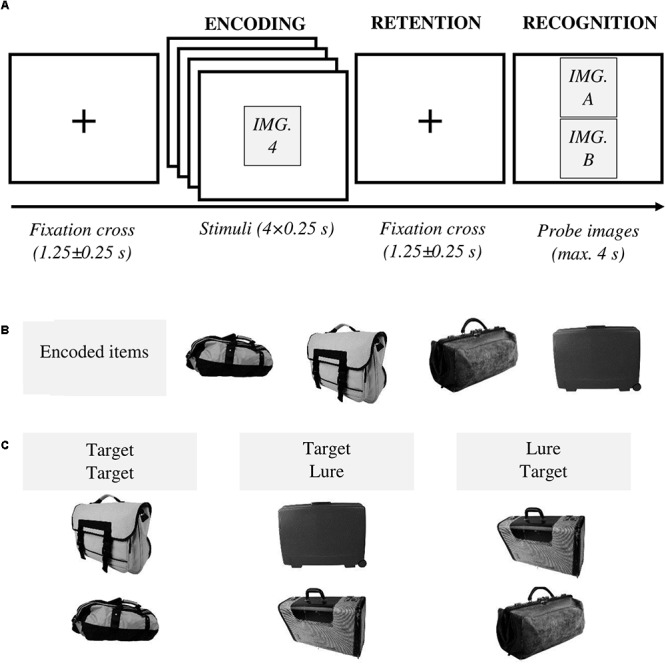
**(A)** Experimental procedure. At the beginning of each trial, a fixation cross was displayed at the center of the screen for 1.25 ± 0.25 s. Next, four images were sequentially presented, centered on the screen, for 0.25 s each. After a retention interval lasting for 1.25 ± 0.25 s, two probe images were shown. Participants had a maximum time of 4 s to decide whether they had previously seen one (or both) image(s) (“yes” answer) or none (“no” answer). Following the “yes” answer, subjects provided additional details identifying the remembered images (not shown in the diagram). Feedback was given after each trial. **(B)** An example of encoded items (targets) for the category “bag.” **(C)** Three types of probes were utilized – i.e., two previously studied items (i.e., target, target), one previously studied item and the lure presented in the lower slot of the presentation screen (i.e., target, lure), one previously studied item, and the lure presented in the upper slot of the presentation screen (i.e., lure, target).

The dataset used in the experiments consisted of 216 sets of commonly seen object categories (e.g., “carpet,” “ball,” and “flower”), each comprising four target images to be encoded (Figure 1B) and a lure image belonging to the category but being never shown during encoding (Figure 1C). All images were converted to grayscale and downsampled to a resolution of 500 × 500 pixels. Pictures were obtained either from the Hemera Photo DVD (Hemera Technologies Inc., Gatineau, Québec) or via Google Images.

### Paradigm

The experimental procedure was as follows (Figure 1A). At the beginning of each trial, a fixation cross was shown for 1.25 ± 0.25 s. In the following encoding interval, four target images were presented sequentially. Each stimulus was displayed in the center of the screen for 250 ms. During the ensuing retention interval, a fixation cross was shown for 1.25 ± 0.25 s and subsequently, two probe images were presented. Participants provided their responses using a gamepad. They had four possible answers to classify both probe images labeled as: *[new item, new item]*, *[old item, new item]*, *[new item, old item]*, and *[old item, old item]* (Table 1). They had a maximum time of 4 s to respond. They were asked to press the “yes” button if they had previously seen one (or both) image(s), meaning at least one image was recognized as old. Whereas “no” corresponded to *[new item, new item]*, i.e., both images were identified as new. Following the “yes” response, subjects were further required to highlight the remembered image(s) using the gamepad joystick as *[old item, new item]*, *[new item, old item]* or *[old item, old item]*. “Yes/no” answer was always provided with the right hand, whereas the joystick was controlled with the left hand. A feedback was given at the end of each trial. Subjects were instructed to respond as quickly and accurately as possible and they were asked not to yield an answer if they were not sure.

**Table 1:** Different answers given by the participants – i.e., *[new item, new item]*, *[old item, new item]*, *[new item, old item]*, and *[old item, old item]* – coupled with the types of probes, allowed for the characterization of the studied three conditions: *correct*, the subject successfully recognized the probes; *false memory*, the lure is presented together with a target – i.e., an encoded item – and the subject mistakenly remembers seeing both images; *error*, the remaining combinations where the subject fails to recognize one or both presented images.

Types of probes	Conditions					
Upper slot	Correct	False memory	Error			
Lower slot
*Target*	*Old item*	*Old item*	*–*	*New item*	*New item*	*–*
*Lure*	*New item*	*Old item*		*Old item*	*New item*	

*Lure*	*New item*	*Old item*	*–*	*Old item*	*New item*	*–*
*Target*	*Old item*	*Old item*		*New item*	*New item*	

*Target*	*Old item*	*–*	*Old item*	*–*	*–*	*New item*
*Target*	*Old item*		*New item*			*New item*
			*OR*			
			*New item*			
			*Old item*			

There were three distinct types of probes (Figure 1C). Lure in the lower slot (target in the upper slot), lure in the upper slot (target in the lower slot) or two targets. No trial included two novel images in the recognition interval. These properties of the probe images were not made explicit to the subjects.

Many STM studies reported higher rates of memory errors for related versus unrelated items, e.g., words (Coane et al., 2007; Atkins and Reuter-Lorenz, 2008; Flegal et al., 2010; Melnik et al., 2017) and faces (Iidaka et al., 2014). In order to induce false memories, the lure was an exemplar semantically related to the studied category.

216 trials of images were randomly presented. Types of probes were randomized and balanced throughout the experiment. Three conditions were defined as follows: *Correct* indicating the successfully answered trials; *false memory*, whenever a lure was misrecognized as previously seen and the concomitant target probe was correctly identified; and *error*, whenever either one or both probed targets were not recognized (Table 1).

The experimental paradigm was implemented in MATLAB^®^ (2014a, The Mathworks, Inc., Natick, MA, United States) using the publicly available Psychophysics toolbox extensions (Brainard, 1997).

### Procedure

Electroencephalography recordings were performed in an acoustically insulated and electrically shielded room. Images were presented foveally on a 21” monitor positioned 90 cm from the subject’s eyes, resulting in a visual angle of 8.41° in both dimensions. Participants completed a preparatory session to become acquainted with the task. The experiment was divided into blocks, each of which composed of ten trials (except for the last block made by six trials). Blocks were separated by self-paced rest breaks in between.

### EEG Recordings

Electroencephalography data were acquired using a 32-channel BrainAmp amplifier (Brain Products, Munich, Germany). Electrodes were mounted in an elastic cap (EasyCap, Herrsching, Germany) and positioned according to the standard international 10–20 system. Mastoids served as reference while ground electrodes were placed on the earlobes. Electrooculogram data were recorded from a pair of electrodes placed, respectively below (for vertical movements) and to the right (for horizontal movements) of the right eye. All impedance levels were kept below 10 kΩ. Data were sampled at the frequency of 1000 Hz.

### Preprocessing and Time-Frequency Analysis

Data analyses were performed using MATLAB with the aid of the open-source Fieldtrip toolbox (Oostenveld et al., 2011) and in-house scripts.

Recordings were bandpass filtered offline using a 4th order Butterworth filter with cut-off frequencies of 0.2 and 100 Hz. Epochs of 3 s, from -1.00 to 2.00 s around the onset of the first stimulus, were extracted and demeaned. Independent component analysis (fast ICA) was applied to remove ocular artifacts. Trials were inspected visually and those still heavily affected by artifacts were discarded. The average number of trials per participant was *M* = 202.39, *SD* = 11.96 (*correct*: *M* = 105, *SD* = 13.98; *error*: *M* = 78.57, *SD* = 14.97; *false memory*: *M* = 18.82, *SD* = 11.09). Time-frequency power estimates were computed using Fourier basis with an Hanning window of 500 ms. Frequencies ranging from 2 to 32 Hz with 2 Hz increments were considered. The time window slid across trials in steps of 50 ms. Power estimates were normalized for each subject and condition, as a percentage of variation from baseline:

$$P_{norm}\;\left(f_{i},\;t_{j}\right)\;=\;100\;\;\times \;\;\frac{P\left(f_{i},\;t_{j}\right)\;-\;P_{baseline}\;\left(f_{i}\right)}{P_{baseline}\;\left(f_{i}\right)}$$

where *f_i_* is the *i^th^* frequency bin and *t_j_* is the *j^th^* time point. *P_norm_* (*f_i_*, *t_j_*) and *P* (*f_i_*, *t_j_*) denote, respectively the normalized power value (reported as a percentage) and the original power estimate for the specific frequency bin *f_i_* and time point *t_j_*^.^ *P_baseline_* (*f_i_*) is the average power value within the baseline for the specific frequency bin *f_i_*. Baseline values were estimated considering all trials, regardless of the condition, within the time interval ranging from -1.00 to -0.30 s prior to the onset of the first stimulus.

### Studied Conditions and Trial Selection for the Oscillatory Analysis

#### Correct and Error

The oscillatory analysis contrasted initially *correct* and *error* conditions. To control signal-to-noise ratio (SNR) differences, an equal number of trials was selected for each condition prior to the estimation of the time-frequency portraits. Accordingly, a pseudorandomized process selected a subset of the condition with a higher number of trials. Trials having response time (RT) lying within one standard deviation from the mean RT of the condition were given priority. When the number of trials having RT within the defined range was not sufficient, trials with RT outside of that range were also considered in order to complete the selection process. With this approach we intended to prioritize oscillatory data corresponding to “typical” behavioral responses.

#### False Memory

After assessing the differences between *correct* and *error* conditions, the focus shifted to *false memory* trials. Notably, channels, frequencies and time intervals relevant to the *correct* and *error* trials were retained for the subsequent analyses (“Cluster Permutation Statistics”). Insufficient number of *false memory* occurrences across participants resulted in low SNR that did not allow for a reliable direct comparison with *correct* and *error* conditions. As a remedy, all *false memory* trials were combined with those of the *error* condition (*error + false memory*), which was then compared with the *correct* condition. Conversely, all *false memory* trials were then merged with the *correct* condition (*correct + false memory*) and the contrast with *error* condition was re-evaluated. False memory trials were added in turn to each condition and statistical results were Bonferroni corrected to account for multiple comparisons. The rationale behind this approach was to use these newly defined conditions, with increased SNR, to highlight pattern similarities of *false memory* with *correct* and *error* conditions.

The trial selection process (analogous to the one described in “Correct and Error”) for the combined conditions – i.e., *correct + false memory* and *error + false memory* – would assure to retain all *false memory* trials.

### Channel Selection and Frequency Bands of Interest for the Encoding Interval

**Figure 2:**
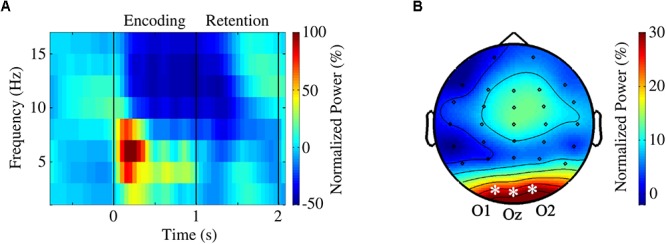
**(A)** Grand average time course averaged over occipital channels O1, Oz, and O2. **(B)** Topographical map of theta activity (4–8 Hz) during encoding [(0,1) s]. A theta power increase, more prominent in occipital regions, was clearly visible.

Given the perceptual nature of our task, occipital channels were anticipated to play a prominent role during the encoding of the visual stimuli. Time-frequency estimates (regardless of the condition) revealed conspicuous occipital power increase within theta band range with simultaneous alpha power decrease immediately after the onset of the first stimulus (Figure 2). Thus, we decided to focus on the analysis of the encoding interval on the occipital channels (O1, Oz, and O2) for theta [(4–8) Hz] and alpha [(10–14) Hz] bands.

### Channel Selection and the Individual Central Frequency (ICF) for the Retention Interval

**Figure 3:**
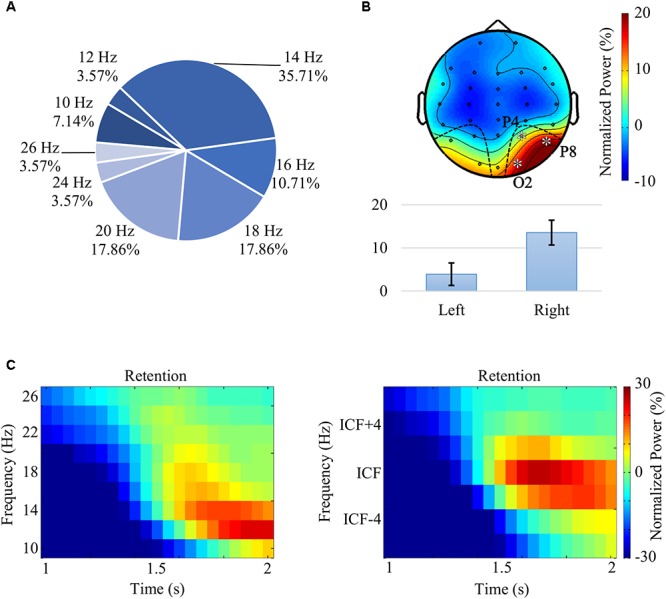
**(A)** Distribution of the individual central frequency (ICF) across all participants. ICFs were spanning across the alpha and beta bands, between 10 and 26 Hz. **(B)** Grand average topography, following the alignment of ICFs, for the frequency range [ICF – 2, ICF + 2] Hz and for the second half of the retention interval [(1.5,2.0) s]. The analysis of the alpha-beta pattern revealed a significant lateralization – *t*(27) = 4.69, *p* < 0.0001, *r* = 0.67 – with power values over right parieto-occipital channels (P4, P8, and O2) that were higher than the ones observed in the contralateral channels (P3, P7, and O1). **(C)** Grand average time-frequency portraits prior (left) and following (right) ICF alignment averaged over the channels O2, P4, and P8.

Visual inspection of time-frequency portraits consistently identified alpha-beta band activity within the second half of the retention interval, i.e., [1.5, 2.0] s, more prominently on the right parieto-occipital channels. A dependent *t*-test contrasting the power difference between the occipital channels of both hemispheres (i.e., power difference between P4, P8, O2, and P3, P7, O1) confirmed the right-sided lateralization of alpha-band power, *t*(27) = 4.69, *p* < 0.0001, *r* = 0.67 (Figure 3B). The central alpha-beta band frequency and associated bandwidth were observed to be subject-dependent – with values ranging from 10 to 26 Hz (Figure 3A). Accordingly, for each participant, we decided to ascertain the individual central frequency (ICF), i.e., the frequency yielding the strongest power increase being consistent with the aforementioned pattern. Consequently, the analysis of the retention interval focused on the channels (P4, P8, and O2) for the frequency range of [ICF – 2, ICF + 2] Hz. Figure 3C show the effect of the ICF alignment on grand average plots.

As the ICF values ranged within the alpha and beta bands, throughout the article we will be referring to that as the “alpha-beta band.” Please note that visual cognition studies such as Waldhauser et al. (2012) and Michalareas et al. (2016) also reported individual frequency peaks spreading over broad alpha and beta band ranges.

### Statistical Analyses of Behavioral Measurements

Statistical analyses pertaining to behavioral data were conducted via IBM SPSS Statistics 22.0 (IBM Corp., Armonk, NY, United States). Friedman’s ANOVA was used to investigate RT differences across conditions as the Kolmogorov-Smirnov test showed that the distribution was non-normal. When required, *post hoc* analyses were realized via Wilcoxon tests and the Bonferroni correction was applied to account for multiple comparisons.

Correlations between RTs (averaged independently from the conditions) and task accuracies (i.e., *correct* response rate) were investigated. Moreover, the relation between the rates of the conditions (i.e., *correct*, *false memory* and *error* rates) was also assessed. As for the RTs, the Kolmogorov-Smirnov test showed that distributions of the rates of conditions were non-normal, thus Spearman’s coefficient was used to estimate the correlations.

### Cluster Permutation Statistics

Statistical analyses of oscillatory data were conducted using the non-parametric cluster-based permutation test (Maris and Oostenveld, 2007), which controls for the multiple comparisons problem. Clusters were defined as two or more contiguous channel-frequency-time triplets, each showing *p* < 0.05 (two-sided dependent samples *t*-test) with respect to the conditions. Cluster-level statistics were computed taking the sum of the *t*-values within each cluster. The reference distribution was approximated by means of the Monte Carlo method with 30000 permutations. The test statistic was defined as the maximum of the cluster-level statistics. A cluster was deemed significant if its Monte Carlo probability exceeded the threshold of 0.025 for each tail when compared to the distribution.

Analysis of the encoding interval [(0,1) s] primarily focused on the elicited pattern, i.e., theta power increase [(4,8) Hz] and alpha power decrease [(10,14) Hz] that was more prominent in the occipital areas (O1, Oz, and O2) (Figure 2B). Furthermore, during the retention interval [(1,2) s], a conspicuous alpha-beta activity increase was observed in the right parieto-occipital channels (P4, P8, and O2) (Figure 3B). Notably, time-frequency estimates of each subject were shifted to align all ICFs and the range of [ICF – 2, ICF + 2] Hz was explored.

As WM studies reported significant correlations between alpha band power values and RTs (e.g., Bonnefond and Jensen, 2012; Obleser et al., 2012; Melnik et al., 2017), we investigated also potential relationship between oscillatory data within the significant clusters and behavioral measures. The correlation was realized via Spearman’s coefficient as the Kolmogorov-Smirnov test determined the distributions of the oscillatory parameters as non-normal.

## Results

### Behavioral Analysis

Participants’ task performance, i.e., *correct* rate, was *M* = 51.10%, *SD* = 5.88% in average. While the *error* rate was *M* = 38.18%, *SD* = 7.64%, *false memory* occurrences rated at *M* = 9.75%, *SD* = 5.30%. Finally, the unanswered trials accounted for the remaining *M* = 0.97%, *SD* = 1.80%.

**Figure 4:**
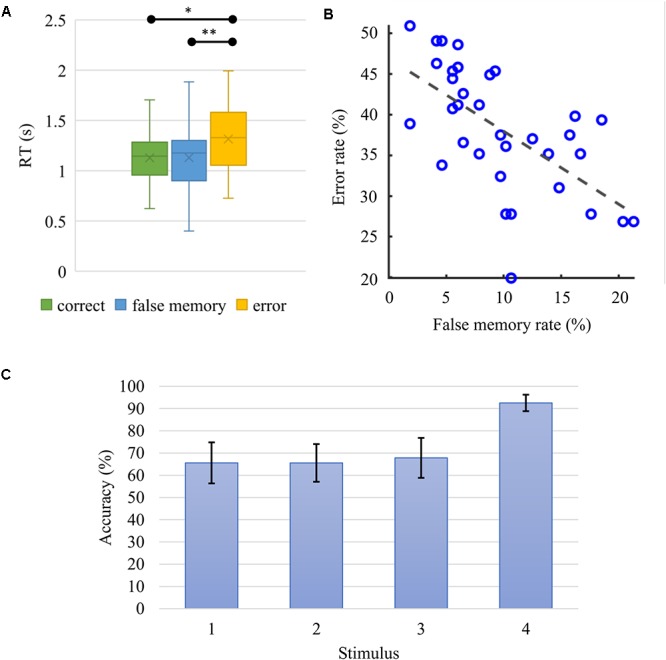
**(A)** Boxplot for the median (IQR) response time (RT) for *correct*, *false memory* and *error* conditions were 1.145 (0.957 to 1.285) s, 1.176 (0.900 to 1.302) s, and 1.330 (1.055 to 1.581) s, respectively. *Correct* and *false memory* conditions recorded significantly faster RT when compared to the *error* condition (^∗^*p* = 0.0000006, ^∗∗^*p* = 0.000175). **(B)** *False memory* and *error* rates correlated negatively (Spearman’s *ρ* = –0.69, *p* = 0.000027). Participants who made more errors had lower *false memory* rate. **(C)** Average accuracy rate and standard deviation as a function of serial position. When probed, the fourth stimulus in the series was recognized with significantly higher accuracy (*p* < 10^-6^) in comparison to the other elements in the sequence.

The RT was significantly influenced by task conditions, χ^2^(2) = 28.294, *p* < 10^-6^. Wilcoxon tests were used to follow-up this finding. Since the Bonferroni correction was applied, statistical significance of effects is reported at the level of *p* = 0.017. Median (IQR) RT for *correct*, *false memory* and *error* conditions were 1.145 [(0.957,1.285)] s, 1.176 [(0.900, 1.302)] s and 1.330 [(1.055,1.581)] s, respectively. There were significant differences between the *error* and *correct* conditions (*Z* = -5.001, *r* = -0.606, *p* = 6 × 10^-7^) and between *error* and *false memory* conditions (*Z* = -3.753, *r* = -0.455, *p* < 0.0002). However, there was no statistically significant difference in RT, when *correct* and *false memory* conditions were compared (*Z* = -0.043, *r* = -0.005, *p* = 0.966) (Figure 4A). A negative correlation was found between *false memory* and *error* rates (Figure 4B, Spearman’s *ρ* = -0.69, *p* = 0.000027). When the recognition rate of each single stimulus was evaluated according to its serial position, we found that the fourth stimulus (*M* = 92.55%, *SD* = 3.71%) was successfully recognized with a rate that was higher (*p* < 10^-6^) than the others (Figure 4C). Please note that the rates were 65.58 ± 9.23, 65.56 ± 8.48, and 67.83 ± 8.98% for the first, second and third items, respectively. As we presented two images during the recognition phase, these rates do not reflect the *global* task performance since subjects need to classify both probes accurately for a response to be considered as *correct* (Table 1). Please note that when only those 28 subjects having EEG data were considered in the behavioral analysis, all aforementioned behavioral results remained statistically valid.

### Oscillatory Analysis

The non-parametric statistical analysis was used to investigate the prominent theta power increase and alpha power decrease observed during the encoding of the stimuli (Figure 2). Conspicuous alpha-beta activity in parieto-occipital regions during the retention interval (Figure 3B) was also assessed.

**Figure 5:**
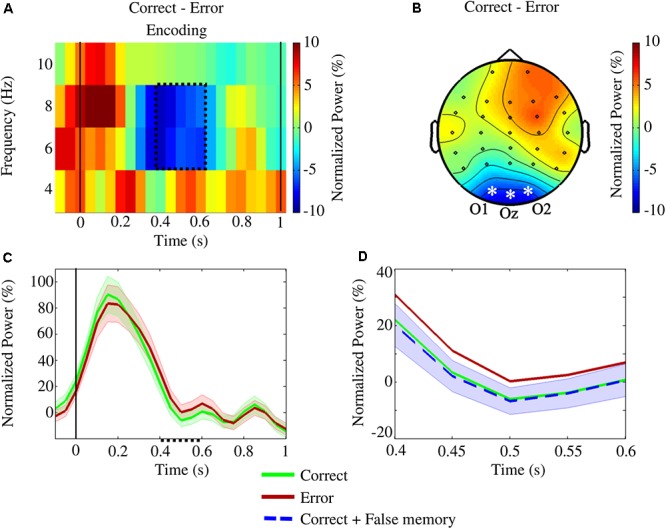
The statistical study of the encoding interval for the conditions *correct* and *error* revealed a significant difference [*p*_(*corrected*)_ = 0.0112] in the upper theta frequencies (6–8 Hz) within [0.40,0.60] s. The analysis focused on the occipital channels O1, Oz, and O2. **(A)** Time-frequency plot of the difference in power between the conditions averaged across the occipital channels. The area enclosed by the dotted line indicates the significant cluster. **(B)** The topographic contrast between the conditions within the cluster (asterisks and labels denote the channels showing significant differences). **(C)** Variation of upper theta power (and standard error), during encoding, averaged over the occipital channels. Within the cluster (dotted line on the x-axis), *error* power values were significantly higher than *correct* ones. **(D)** Variation of upper theta power (and standard error) within the cluster: when *false memory* trials were added to the *correct* condition, a significant difference was still observed on the channels O1, Oz, and O2 [*p*_(*corrected*)_ = 0.0200] at [6,8] Hz within [0.40,0.55] s – inversely, no significant cluster was observed when *false memory* trials were added to the *error* condition.

The cluster-based permutation test revealed a significant difference between *correct* and *error* conditions [*p*_(*corrected*)_ = 0.0112] in the encoding interval [(0.40, 0.60)] s, with *error* eliciting higher theta power [(6,8) Hz] in all three occipital channels (Figure 5A–C). When trials from *false memory* and *correct* were grouped together and compared with *error*, a significant difference was still identified for [6,8] Hz and [0.40,0.55] s [*p*_(*corrected*)_ = 0.0200], with *error* showing higher theta activity (Figure 5D). Inversely, no significant cluster was found when trials from *false memory* were added to the *error* condition and differences with *correct* were reassessed. There was no statistically significant difference between conditions regarding the alpha band power in the occipital channels during the encoding interval.

**Figure 6:**
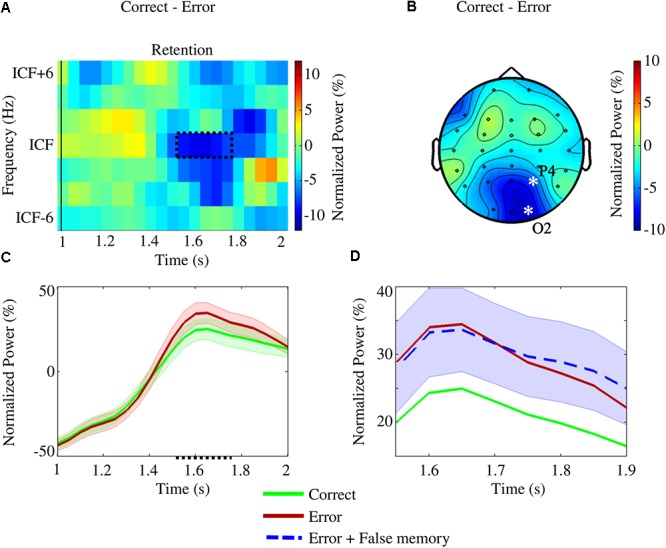
The analysis of the retention interval investigated differences between *correct* and *error* conditions in the right parieto-occipital channels (P4, P8, and O2) considering the subjects’ individual central frequencies (ICFs). A significant cluster [*p*_(*corrected*)_ = 0.0203] was found for the ICF in O2 [at (1.50,1.75) s] and P4 [at (1.70,1.80) s]. **(A)** Time-frequency portrait of the difference between the conditions for the representative channel O2. The region enclosed by the dotted line highlights the significant cluster. **(B)** Topographic pattern of the difference between the conditions within the discovered cluster (asterisks and labels denote the channels showing the significant difference). **(C)** Variation of ICF power (and standard error) within the retention interval for the representative channel O2. Inside the significant region (dotted line on the x-axis), *error* power values were higher when compared to the *correct* ones. **(D)** Variation of ICF power (and standard error) within the cluster for the representative channel O2: when *false memory* and *error* trials were merged, a significant difference [*p*_(*corrected*)_ = 0.0393] was still measured for the ICF in O2 [at (1.55,1.90) s] and P4 [at (1.75,1.80) s] – inversely, no significant cluster was observed when *false memory* trials were added to the *correct* condition.

Following the frequency shift for aligning the subjects’ ICF, the analysis within the retention interval revealed a significant difference between *correct* and *error* conditions [*p*_(*corrected*)_ = 0.0203], with *error* showing higher power values than those for *correct* in O2 (at the ICF, [1.50,1.75] s), and P4 [at the ICF, (1.70,1.80) s; Figure 6A–C]. The addition of trials from *false memory* to the *error* condition produced, contrary to the encoding interval, a significant cluster [*p*_(*corrected*)_ = 0.0393], when compared to *correct*, in O2 [at the ICF, (1.55,1.90) s], and P4 [at the ICF, (1.75,1.80) s; Figure 6D]. However, no significant difference was found out when trials from *false memory* and *correct* were merged and compared with *error*.

**Figure 7:**
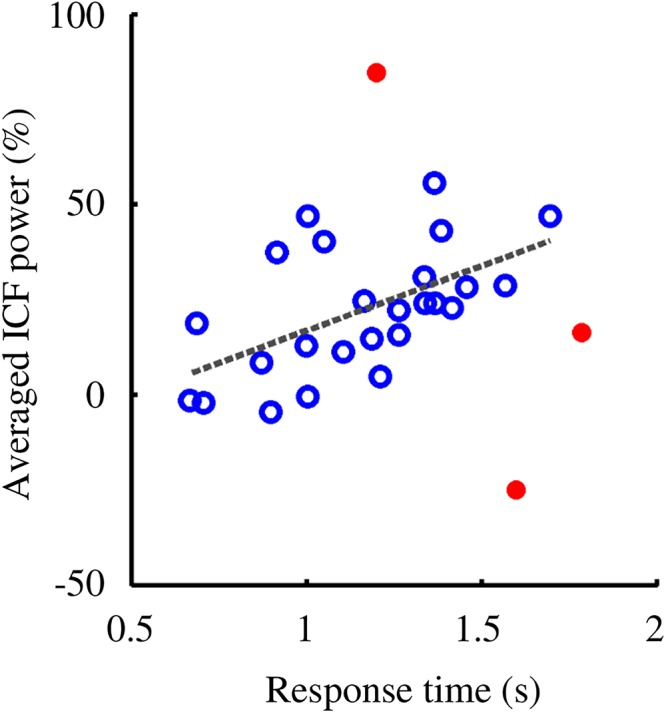
RT and ICF power showed a positive correlation (Spearman’s *ρ* = 0.60, *p* = 0.0020). Participants with higher ICF power responded, on average, slower to the probes. Subjects included in the correlation analysis are denoted by empty circles. Outliers, highlighted with filled circles, were removed from the analysis as their values fell out of the 95% confidence interval.

Investigation of the relation between behavioral and oscillatory properties revealed a positive correlation between average ICF power (estimated within the significant cluster of the retention interval) and mean RT (Figure 7, Spearman’s *ρ* = 0.60, *p* = 0.002; three subjects were excluded as their parameters fell out of the 95% confidence interval).

## Discussion

This study explored the role of cortical brain oscillations in memory by analyzing behavioral and EEG data of healthy volunteers performing a challenging VWM task. Specifically, we tested whether changes in oscillatory activity during encoding and retention of the sequentially presented four images can predict the quality of memory formation.

We found that theta oscillations during encoding of successful memories exhibited power values in occipital channels that were significantly lower when compared to the incorrect ones. In the following retention interval, errors elicited alpha-beta (ICF) power values higher than those of correct answers in right parieto-occipital channels. Further, we investigated the oscillatory properties of false memory over short-term periods. Our analysis suggested pattern similarities in theta band during encoding between false and successful memories in occipital channels with power values that were lower than the erroneous ones. Conversely, during the retention interval, *false memory* and *error* showed a similar alpha-beta band (ICF) pattern with power levels in right parieto-occipital channels higher than those of *correct* responses.

Investigation of the relationship between behavioral and oscillatory properties revealed a positive correlation between average ICF power (within the significant cluster of the retention interval) and mean RT. The behavioral analysis further revealed how RTs of the *error* condition were significantly longer than those of *correct* and *false memory*. The negative correlation between *false memory* and *error* rates showed that subjects who performed poorly had lower rates of *false memory* responses.

### Correct and Error

Our results showed an association between lower theta power values and successful encoding, while for errors, a poor sequential encoding of the stimuli was reflected by higher theta power values. Involvement of theta oscillations in WM tasks has been widely reported (e.g., Kahana et al., 2001; Jacobs and Kahana, 2010). The observed alpha power decrease accompanied by a simultaneous theta power increase was consistent with the pattern associated with intentional encoding (Mölle et al., 2002) and memory formation (Klimesch et al., 1997b; Osipova et al., 2006).

Theta power increase in occipital channels may reflect the formation of cell assemblies. They are reported to be functionally related to processes such as feature binding and formation of memories (Singer and Gray, 1995; Bastiaansen and Hagoort, 2003; Buzsáki, 2010). Cognitive mechanisms during encoding and retrieval periods vary considerably with respect to the task specificity, hence altering the corresponding oscillatory processes responsible for proper memory formation (Hanslmayr and Staudigl, 2014). Various brain oscillatory studies linked successful memory performances to significant increases in theta band power during encoding (Sederberg et al., 2003; Osipova et al., 2006; White et al., 2013). In contrast, lower levels of theta power corresponding to successful memory formation were also observed depending on the brain region of interest and the time of encoding (Sederberg et al., 2006; Guderian et al., 2009; Burke et al., 2013). Moreover, subbands of theta activity may show opposite tendencies of power levels for correct encoding, i.e., higher power for slow theta ∼ 3Hz and lower power for fast theta ∼8 Hz (Lega et al., 2012). This is in line with our findings as the prominent theta activity was observed in the upper range of 6–8 Hz.

Oscillatory analysis revealed a further difference in the alpha-beta band during the retention interval where the *error* condition elicited higher power values than the *correct* one. The frequency range of alpha-beta band activity (i.e., the frequency band where the observed parieto-occipital pattern was more prominent) varied across subjects and therefore the central band frequency was determined individually as ICF values spread diversely within the range of [10–26 Hz]. Alpha peak frequency in posterior regions was shown to be subject-dependent and to increase with higher cognitive load (Haegens et al., 2014). The observed oscillatory activity during the retention interval is in line with the alpha band inhibition-timing hypothesis (Klimesch et al., 2007). Accordingly, the alpha-beta band activity may reflect the suppression of the visual input via disengagement of the visual dorsal pathway (Vanni et al., 1997; Jensen et al., 2002; Cooper et al., 2003; Jokisch and Jensen, 2007; Tuladhar et al., 2007; Scheeringa et al., 2009).

In our study, alpha-beta band activity was lateralized to the right parieto-occipital channels. Among studies reporting lateralization of alpha activity in the posterior regions, Bonnefond and Jensen (2012) showed the left-lateralized alpha power enabling the suppression of anticipated distractors (symbols or letters). Alpha oscillations were also found out to be modulated by visual attention (Worden et al., 2000). More specifically, prior to the onset of the stimulus, alpha power increase was observed over the occipital regions, ipsilateral to the cued direction of attention, aiming to suppress irrelevant stimuli presented over a to-be-ignored location (Leenders et al., 2018). In a subsequent retention interval, an increase in alpha power contralateral to the irrelevant stimulus was related to WM maintenance processes responsible for suppressing the distractors. Both Jensen et al. (2002) and Scheeringa et al. (2009) reported a right lateralization in the alpha band range during the maintenance phase of a verbal WM task. These studies indicate the excitatory/inhibitory roles of alpha band. As our study used complex visual stimuli centrally located on the screen, the lateralization cannot be explained by shifts in visual attention or by factors concerning the spatial location of the stimuli.

The oscillatory power asymmetry taken with the inhibitory function of alpha band suggests an active role of the contralateral regions belonging to the left hemisphere during the retention interval in our study. Parra et al. (2014) reported the engagement of left posterior cortical areas during maintenance in VWM of multi-feature objects. They identified BOLD activity in the left fusiform gyrus (near the LOC) and left parietal cortex related to the maintenance of the binding of visual features. In a transcranial alternating current stimulation (tACS) study, Tseng et al. (2016) demonstrated the recruitment of similar regions of left temporal and parietal cortex, when binding of perceptual features is realized within VWM.

Higher levels of alpha-beta activity recorded for *error* may be indicative of an inefficient allocation of WM storage. This view is supported by the positive correlation of inhibitory alpha power and memory load reported during WM maintenance over task-irrelevant regions (Jensen et al., 2002; Tuladhar et al., 2007). The positive correlation between alpha-beta (ICF) power in retention and RTs further supports the idea that higher alpha activity values may reflect an inefficient WM performance. Similarly, a positive correlation between alpha power and RT in the retention interval was reported by Roux et al. (2012).

We would like to note that the *error* condition includes also trials where either of the two target probes was recognized correctly. As a poor encoding sequence may include stimuli that were encoded properly, the oscillatory contrast between *correct* and *error* conditions likely weakens. However, this would not affect the character of the reported oscillatory markers distinguishing erroneously and correctly encoded trials in essence.

### False Memory

As “memory is often accurate” (Slotnick and Schacter, 2004) typically, a lower rate is expected for false and erroneous memories compared to the correct responses. Induction of high rates of false memory responses is especially challenging for paradigms with short durations. Some studies such as Atkins and Reuter-Lorenz (2011) and Melnik et al. (2017) have used strategies of extra distractors of math questions engaging cognitive faculties in order to increase false memory rates. In those studies, there were still no adequate number of trials, allowing comparison with the correctly answered ones. Despite the cognitively demanding task that involves fast-paced encoding and two different probes to be answered, an average false memory rate of 9.75 ± 5.30% could be obtained in our study.

Due to insufficient number of *false memory* trials, we were not able to make a direct statistical comparison with the other conditions. However, we assessed potential similarities of *false memory* with *correct* and/or *error* by adding in turn *false memory* trials to *correct* (contrasted with *error*) and *error* (contrasted with *correct*) conditions. The balanced number of trials and the increased SNR for the conditions enabled us to perform meaningful comparisons.

Our analysis suggested similarities regarding the encoding pattern between false and successful memories. This observation upheld the idea that proper encoding is a prerequisite for associative false memories. The negative correlation observed between *error* and *false memory* rates is consistent with the idea that even for the case of STM, false memories may be a byproduct of adaptive processes allowing an efficient functioning of the human memory system (Schacter et al., 2011).

While false memories shared similar oscillatory characteristics with successful memories during encoding, they also had similar tendencies with *error* responses, though only within the retention interval. That is, common errors and false memories both showed higher alpha-beta power than that of *correct* responses. Whereas high levels of power for errors indicates an inefficient use of WM storage, our results imply that this may not be the case for false memories. As posterior alpha power may increase with WM load during retention (Jensen et al., 2002; Tuladhar et al., 2007), it possibly reflects extra load caused by pre-existing semantic associations (Koutstaal et al., 2003) and/or prototypical perceptual features (Gutchess and Schacter, 2012) elicited by the encoded stimuli.

Please note that, the scope of our task comprised all encoded items within the same category. Different category items would modify the encoding mechanism altogether, which would increase the variability between the conditions. Moreover, that would likely lead to insufficient number of erroneous trials to be compared in oscillatory analysis as unrelated probe items are notoriously recognized with much higher rates. A series of studies assessed the effect of relatedness on STM and showed that rates of false recognitions for related lures were significantly higher than those of unrelated ones (e.g., Coane et al., 2007; Atkins and Reuter-Lorenz, 2008; Flegal et al., 2010; Iidaka et al., 2014; Melnik et al., 2017).

## Conclusion

In this study, we aimed to identify oscillatory markers distinguishing successful and erroneous visual memories and investigate oscillatory properties characterizing the phenomenon of short-term false memory. The theta power increase observed in occipital channels may reflect the formation of cell assemblies linked to feature binding or formation of memories. We demonstrated how theta power could index the quality of encoding. Our results suggested that the smaller theta power observed for correct responses correspond to an optimal encoding. On the contrary, the inefficient encoding of erroneous trials was accompanied with higher theta power values. False memories revealed a similar trend and contrasted with the pattern characterizing other memory errors. Thus, a proper encoding strategy may leave participants more vulnerable to false memories.

The inhibitory alpha-beta power observed in the retention interval was higher for erroneous memories suggesting that errors are characterized by an inefficient allocation of WM storage. On the other hand, higher alpha-beta power levels for false memories indicate the failure to manage the extra load induced by the encoded stimuli. The negative correlation between the rates of *error* and *false memory* further implies that the latter is an undesired outcome of adaptive processes responsible for the efficient functioning of memory.

## Data Availability Statement

The raw data supporting the conclusions of this manuscript will be made available by the authors, without undue reservation, to any qualified researcher.

## Ethics Statement

This study was carried out in accordance with the recommendations of the Middle East Technical University (METU) ethics committee (http://www.metu.edu.tr/code-ethics-core-values). The protocol was approved by the Middle East Technical University (METU) ethics committee. All subjects were informed about the experimental procedure and provided written informed consent prior to data collection in accordance with the Declaration of Helsinki.

## Author Contributions

IM and TÖ co-designed the study and wrote the manuscript. TÖ conceived the original idea behind the experiments. IM collected the data and carried out the analyses.

## Conflict of Interest Statement

The authors declare that the research was conducted in the absence of any commercial or financial relationships that could be construed as a potential conflict of interest.
